# The Association between Emergency Department Length of Stay and In-Hospital Mortality in Older Patients Using Machine Learning: An Observational Cohort Study

**DOI:** 10.3390/jcm12144750

**Published:** 2023-07-18

**Authors:** Lijuan Wu, Xuanhui Chen, Anna Khalemsky, Deyang Li, Taoufik Zoubeidi, Dominique Lauque, Mohammed Alsabri, Zoubir Boudi, Vijaya Arun Kumar, James Paxton, Dionyssios Tsilimingras, Lisa Kurland, David Schwartz, Said Hachimi-Idrissi, Carlos A. Camargo, Shan W. Liu, Gabriele Savioli, Geroge Intas, Kapil Dev Soni, Detajin Junhasavasdikul, Jose Javier Trujillano Cabello, Niels K. Rathlev, Karim Tazarourte, Anna Slagman, Michael Christ, Adam J. Singer, Eddy Lang, Giovanni Ricevuti, Xin Li, Huiying Liang, Shamai A. Grossman, Abdelouahab Bellou

**Affiliations:** 1 Institute of Sciences in Emergency Medicine, Department of Emergency Medicine, Guangdong Provincial People’s Hospital (Guangdong Academy of Medical Sciences), Southern Medical University, Guangzhou 510080, China; wulj1989@163.com (L.W.);; 2 Medical Research Institute, Guangdong Provincial People’s Hospital (Guangdong Academy of Medical Sciences), Southern Medical University, Guangzhou 510080, China; 3 Medical Big Data Center, Guangdong Provincial People’s Hospital (Guangdong Academy of Medical Sciences), Southern Medical University, Guangzhou 510080, China; 4 Management Department, Hadassah Academic College, Jerusalem 91010, Israel; 5 Department of Statistics, College of Business and Economics, UAE University, Al Ain 1555, United Arab Emirates; 6 Department of Emergency of Medicine, Beth Israel Deaconess Medical Center, Teaching Hospital of Harvard Medical School, Boston, MA 02115, USA; 7 Department of Emergency Medicine, Purpan Hospital and Toulouse III University, 31300 Toulouse, France; 8 Department of Pediatrics, Brookdale University Hospital and Medical Center, 1 Brookdale Plaza, Brooklyn, NY 11212, USA; 9 Department of Emergency Medicine, Dr Sulaiman Alhabib Hospital, Dubai 2542, United Arab Emirates; 10 Global Network on Emergency Medicine, Brookline, MA 02446, USA; 11 Department of Emergency Medicine, Wayne State University School of Medicine, Detroit, MI 48201, USA; 12 Department of Family Medicine & Public Health Sciences, Wayne State University School of Medicine, Detroit, MI 48201, USA; 13 Department of Medical Sciences, Örebro University, 70182 Örebro, Sweden; 14 Information Systems Department, Graduate School of Business Administration, Bar-Ilan University, Ramat-Gan 529002, Israel; 15 Department of Emergency Medicine, Ghent University Hospital, 9000 Ghent, Belgium; 16 Department of Emergency Medicine, Massachusetts General Hospital, Harvard Medical School, Boston, MA 02114, USA; 17 Emergency Department, IRCCS Fondazione Policlinico San Matteo, 27100 Pavia, Italy; 18 Department of Critical Care, General Hospital of Nikaia Agios Panteleimon, 18454 Athens, Greece; 19 Jai Prakash Narayan Apex Trauma Center, Ring Road, New Delhi 110029, India; 20 Department of Medicine, Faculty of Medicine Ramathibodi Hospital, Mahidol University, Bangkok 10400, Thailand; 21 Intensive Care Unit, Hospital Universitari Arnau de Vilanova, 25198 Lleida, Spain; 22 Department of Emergency Medicine, University of Massachusetts Medical School, Baystate, Springfield, MA 01199, USA; 23 Department of Health Quality, University Hospital, Hospices Civils, 69002 Lyon, France; 24 Department of Emergency Medicine, University Hospital, Hospices Civils, 69002 Lyon, France; 25 Division of Emergency and Acute Medicine, Campus Virchow Klinikum and Charité Campus Mitte, Charité Universitätsmedizin, 10117 Berlin, Germany; 26 Department of Emergency Medicine, 6000 Lucerne, Switzerland; 27 Department of Emergency Medicine, Renaissance Scholl of Medicine at Stony Brook University, Stony Brook, NY 11794, USA; 28 Department of Emergency Medicine, Emergency Medicine Cumming School of Medicine, University of Calgary, Alberta Health Services, Calgary, AB T2N 1N4, Canada; 29 Emergency Medicine, School of Pharmacy, University of Pavia, 27100 Pavia, Italy; 30 Department of Emergency Medicine, Guangdong Provincial People’s Hospital (Guangdong Academy of Medical Sciences), Southern Medical University, Guangzhou 510080, China

**Keywords:** emergency department, in-hospital mortality, length of stay, boarding time, machine learning, older adults

## Abstract

The association between emergency department (ED) length of stay (EDLOS) with in-hospital mortality (IHM) in older patients remains unclear. This retrospective study aims to delineate the relationship between EDLOS and IHM in elderly patients. From the ED patients (n = 383,586) who visited an urban academic tertiary care medical center from January 2010 to December 2016, 78,478 older patients (age ≥60 years) were identified and stratified into three age subgroups: 60–74 (early elderly), 75–89 (late elderly), and ≥90 years (longevous elderly). We applied multiple machine learning approaches to identify the risk correlation trends between EDLOS and IHM, as well as boarding time (BT) and IHM. The incidence of IHM increased with age: 60–74 (2.7%), 75–89 (4.5%), and ≥90 years (6.3%). The best area under the receiver operating characteristic curve was obtained by Light Gradient Boosting Machine model for age groups 60–74, 75–89, and ≥90 years, which were 0.892 (95% CI, 0.870–0.916), 0.886 (95% CI, 0.861–0.911), and 0.838 (95% CI, 0.782–0.887), respectively. Our study showed that EDLOS and BT were statistically correlated with IHM (*p* < 0.001), and a significantly higher risk of IHM was found in low EDLOS and high BT. The flagged rate of quality assurance issues was higher in lower EDLOS ≤1 h (9.96%) vs. higher EDLOS 7 h <t≤ 8 h (1.84%). Special attention should be given to patients admitted after a short stay in the ED and a long BT, and new concepts of ED care processes including specific areas and teams dedicated to older patients care could be proposed to policymakers.

## 1. Introduction

Emergency departments (ED) are the first healthcare settings that patients with acute illness encounter prior to admission to the hospital. The imbalance between the demands of ED patients and the availability of ED resources to provide emergency care has caused overcrowding in the ED, which has been identified as one of the main factors compromising timely and efficient care [1]. ED/hospital crowding has become a significant public health problem across the globe. Boarding and overcrowding have been intensified during the COVID-19 pandemic [2].

The time elapsed between ED arrival and ED discharge is defined as ED length of stay (EDLOS). Prolonged EDLOS is believed to be one of the major factors associated with ED overcrowding and affects clinical outcomes adversely [3]. However, the definition of prolonged EDLOS varies across countries, for example, prolonged ED visits have been defined as >4 h in the United Kingdom, >6 h in Canada and the US, and >8 h in Australia [4,5]. Boarding time (BT) is defined as the time spent between the ED decision to admit the patient to the hospital and ED departure time and is considered an important contributor to the EDLOS. Prolonged BT will occupy resources in the ED and potentially affect the outcomes of other patients [6,7].

In our recent meta-analysis and systematic review, we found that there was an association between EDLOS and IHM for patients with EDLOS below 3 h in non-ICU-admitted ED patients [8]. Mohr et al. [3] found that prolonged BT in the ED, thus prolonging EDLOS, is associated with worse clinical outcomes including mortality, particularly in critically ill patients. Although there is a significant association between crowding and EDLOS, the relationship between EDLOS and in-hospital mortality (IHM) remains unclear.

Given the lack of evidence, additional studies are needed to examine the association between EDLOS and IHM using real-world data. This study attempts to fill the gap by finding evidence of the relationship between EDLOS and IHM, which could potentially help to improve patient experiences and outcomes, relieve the stress of ED healthcare providers, create a better working environment, and support hospitals’ managerial decisions and policy making. The aim of this study was to examine the association of EDLOS with IHM among older patients who were admitted to the hospital from the ED.

## 2. Materials and Methods

### 2.1. Study Population

**Figure 1:**
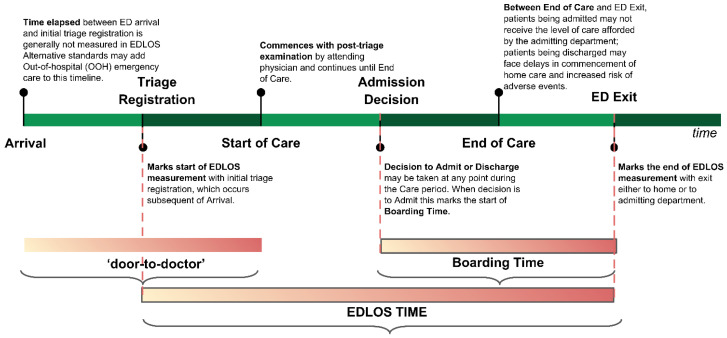
Definition of time spent in the emergency department related to each segment of the care process. This figure is obtained from an open-access article distributed under the Creative Commons Attribution License, which permits unrestricted use, distribution, and reproduction in any medium, provided the original work is properly cited [8].

**Figure 2:**
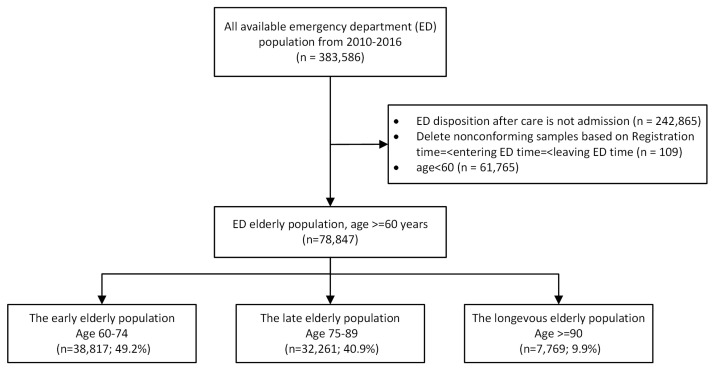
Sample selection process.

All patients admitted to the ED (57,000 visits per year, 150 nurses, 64 senior doctors, 39 residents) of an urban academic tertiary care medical center in the US between January 2010 and December 2016 were collected. The ED has an observation unit which is considered an in-hospital unit. The IHM analyzed in our study includes the patients who died in the observation unit. The EDLOS defined in Figure 1 does not include the time spent in the observation unit. This study was approved by the institutional review board of Beth Israel Deaconess Medical Center, Boston, MA (Approval Number: 2016P-000439). From a total of 383,586 encounters, we excluded those samples who were (a) young patients (age at visit <60 years) (n = 61,765), (b) not admitted to the hospital after an ED visit (i.e., discharge) (n = 242,865), or (c) experienced an unreasonable relative order of several time records (time of triage registration, time of the start of care, time of the disposition decision, and the time at ED departure) to determine the EDLOS (n = 109). For example, the ED entry time was later than the ED exit time. The final retrospective cohort contained 78,847 elderly encounters and was stratified into three age groups based on the World Health Organization criteria for the classification of older persons: the early elderly group (age 60–74 years; n = 38,817; 49.2%), the late elderly group (age 75–89 years; n = 32,261; 40.9%), and the longevous elderly group (age ≥ 90 years; n = 7769; 9.9%) (see Figure 2).

### 2.2. Data Collection and Data Processing

For each ED encounter of the cohort, we extracted demographic and clinical features recorded in the electronic medical records (EMR), including age, gender, race, language (English and non-English), health insurance categories, mode of transport (such as walk-in, ambulance, and helicopter), level of triage acuity score measured using a 5-point scale (i.e., level 1—resuscitation, level 2—emergency, level 3—urgent, level 4—less urgent, and level 5—nonurgent), principal diagnosis codes (i.e., ICD-9 or ICD-10 codes), ED disposition after care (such as ICU and non-ICU), patient medical histories using the Charlson Comorbidity Index, ED waiting time (between triage registration and the start of care), ED boarding time (between the admission decision and departure from the ED), and the EDLOS time (from ED arrival until the patient left the ED) (see Figure 1). We also extracted the quality assurance issues (QAI) flagged by any healthcare provider when they suspected a patient safety event (PSE), defined as a negative health outcome suspected to be related to a medical error during ED care [9]. The outcome of interest was the death during hospitalization. For each age group, variables missing in more than 99% of the population were excluded to reduce the EMR variable dimension. The interquartile range (IQR) technique [10] was used to remove outliers. Specifically, the upper and lower limits were set to 5 times the IQR, and any observation beyond the limits would be considered a potential outlier. One-hot encoding (or dummy variable processing) was used to turn the categorical variables into a binary vector representation.

### 2.3. Experimental Methodology

This research mainly explored four machine learning prediction models, i.e., Logistic Regression, Random Forest [11], eXtreme Gradient Boosting (XGBoost) [12], and Light Gradient Boosting Machine (LightGBM) [13]. LightGBM contains two novel techniques, Gradient-based One-Side Sampling and Exclusive Feature Bundling for processing a large number of data samples and features, respectively, which becomes a highly efficient gradient boosting decision tree in terms of computational speed and memory consumption. In addition, a popular predictive interpretation technique, the game theory-inspired Shapley Additive exPlanations (SHAP) [14], was applied to explain the predictive model at the individual patient and population level. A positive SHAP value means that the presence of the variable increases the likelihood of the adverse outcome for this sample. A negative SHAP value suggests that the presence of the variable decreases the likelihood of the adverse outcome for a particular patient. If a SHAP value is close to 0, this suggests that the model does not consider the variable relevant to estimating the likelihood.

### 2.4. Diagnosis Subgroup Analysis

Due to differences in the distribution of EDLOS and BT among different diagnosis populations, it was necessary to conduct subgroup analysis on different main diagnoses to verify the association between EDLOS/BT and IHM. We extracted the main diagnostic information of each patient, which is represented by ICD9/ICD10 codes, and each type was further divided into 3-digit/3-char, 4-digit/4-char, and 5-digit/5-char codes. In order to unify the diagnostic grouping, as shown in Supplementary Figure S1, we first unified the different digit/char codes into 3 digits/chars, then mapped them to their respective ICD9/ICD10 main categories, and finally unified the code categories of ICD9 and ICD10.

### 2.5. Statistical Analysis

Continuous variables were presented as a mean (standard deviation, SD) for normal distribution or a median (interquartile range, IQR) for non-normal distribution, whereas categorical data were presented as a frequency (percentage). For missing categorical data, a value of 0 was set as a separate category, while for numerical data, missing values were not imputed because the used tree-based machine learning models (e.g., LightGBM) can handle missing values and the optimal null value splitting direction was obtained by automatic learning based on improvement in training performance. The t test or Kruskal–Wallis test was used to test group mean differences for continuous variables, and the Chi-square test, or Fisher’s exact test, was used for categorical variables to check the association. Since the dataset was large enough, 10-fold cross-validation (CV) was applied to evaluate the effectiveness of machine learning models, the original samples were randomly divided into 10 subsamples, where one subsample (i.e., 10%) was retained as the validation data for testing the classifier, and the remaining 9 subsamples (i.e., 90%) were used as training data. Furthermore, the CV process was then repeated 10 times, with each of the 10 subsamples used as the test data only once. The 10 results from the folds were then averaged to produce a single performance estimation. The relationship between EDLOS/BT and IHM was evaluated by stratified analyses using multivariable logistic regression models [odds ratio (OR) and 95% confidence interval (CI)]. The area under the receiver operator characteristic curve (AUROC) and the 95% bootstrapped CI were used to compare the overall prediction performances. Delong’s test [15] (a nonparametric test) was used to calculate the statistical significance for comparing AUROCs of two or more correlated ROC curves. Two-tailed p < 0.05 denoted statistical significance for all comparisons. Data processing and analysis were performed using Python 3.7 with open-source python packages (e.g., “xgboost”, “lightgbm” and “shap”) and scikit-learn libraries.

## 3. Results

Of the 78,847 encounters meeting the inclusion criteria, IHM occurred in 2975 (3.8%). As shown in Table 1, the median (Q1, Q3) of EDLOS and BT in the elderly population were 366 (271, 495)and 143.0 (104, 219) minutes, respectively. IHM increases with age and is distributed in each group as follows: 2.7% in the 60–74 age group, 4.5% in the 75–89 age group, and 6.3% in the ≥90 age group. The proportion of males decreased with age (51.7% in the 60–74 age group vs. 33.8% in the ≥90 age group, p< 0.001). The IQR increased with age (p< 0.001), i.e.ƒ, 882 (2.3%), 859 (2.7%) and 219 (2.8%) for the 60–74, 75–89, and ≥90 age groups, respectively. Charlson Comorbidity Index scores were higher in early elderly patients than in longevous elderly patients (e.g., for Charlson score >2, 6.0% vs. 1.4%). The walk-in mode of transport was more frequent in the early elderly patients (48.6%), while the ambulance mode was more frequent in the late elderly (59.4%) and longevous elderly patients (70.1%). QAIs were observed in 2.3%, 2.7%, and 2.8% of the 60–74, 75–89, and ≥90 age groups, respectively.

Supplementary Table S1 illustrates the characteristics of patients according to IHM (survivors and non-survivors). Supplementary Table S2 shows the distribution of the variables for older patients admitted to ICU versus those not admitted to ICU. Supplementary Table S3 compares the characteristics of survivors and non-survivors in the low EDLOS group (EDLOS < 300 min) and the high EDLOS group (EDLOS ≥ 300 min) in the entire elderly population, where the percentage of non-survivors in the low-EDLOS population is higher than in the high-EDLOS population (i.e, 6.1% vs. 2.6%, p< 0.001), and the incidence of QAI in non-survivors of low-EDLOS population is higher than in the high-EDLOS population (i.e., 28.7% vs. 17.9%, p< 0.001).

**Figure 3:**
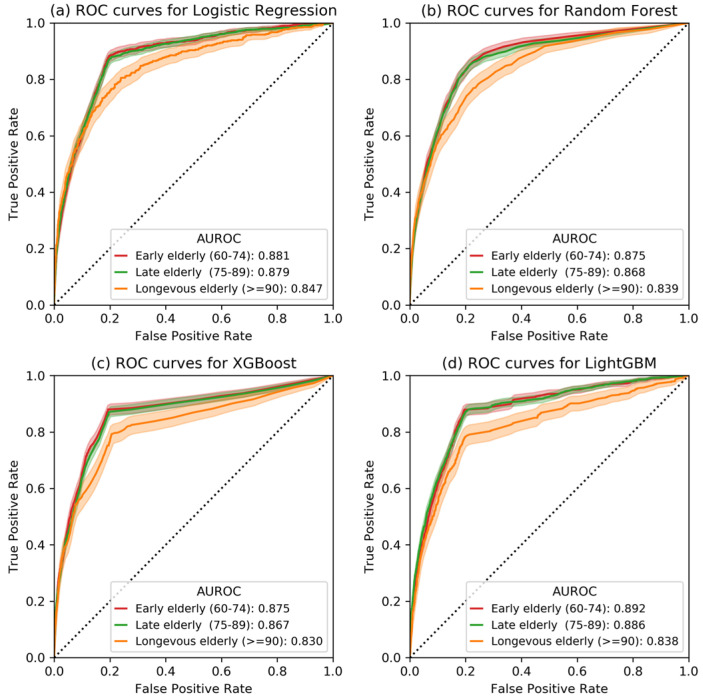
The receiver operating characteristic curves of four prediction models (i.e., Logistic Regression, Random Forest, XGBoost, and LightGBM) for three age groups. The dark line represents the mean ROC curves, and the light area represents the corresponding 95% confidence interval.

Figure 3 shows the receiver operator characteristic (ROC) curves of four machine learning models in the three age subgroups. From this figure, we observed that the predictability of IHM decreased as age increased (p < 0.001, Delong’s test). Supplementary Table S4 illustrates the AUROC and corresponding 95% CI for IHM prediction in four age groups based on four machine learning models (i.e., logistic regression, random forest, XGBoost, and LightGBM). For example, AUROCs of the LightGBM model for ages 60–74, 75–89, and ≥90 years were 0.892 (95% CI, 0.870–0.916), 0.886 (95% CI, 0.861–0.911), and 0.838 (95% CI, 0.782–0.887), respectively. Since the tree-based LightGBM model outperformed the other three models, the LightGBM was chosen as the final predictive classifier for the SHAP explainer.

**Figure 4:**
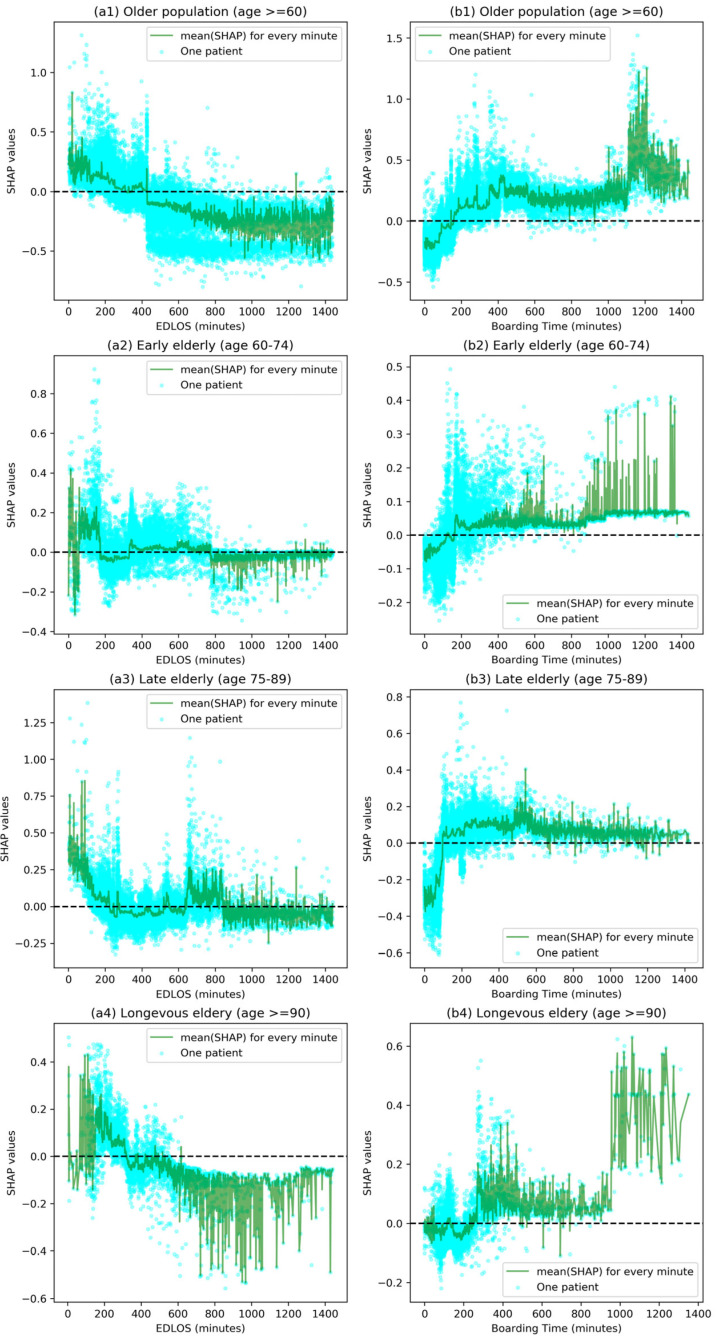
Effect of varying emergency department length of stay (EDLOS, in minutes) and boarding time (BT, in minutes) on in-hospital mortality (IHM) for the whole older population and three age subgroups based on the SHAP method. These plots show the IHM risk for a given EDLOS/BT value for all samples. Each cyan dot represents a patient. The higher the SHAP value of EDLOS/BT, the higher risk of IHM due to this feature value. The dark green line represents the average risk of all samples with a given EDLOS/BT value (in minutes).

Figure 4 shows the SHAP values corresponding to the specific EDLOS and BT for each patient in the whole older population (age ≥ 60 years) and three age subgroups, and the mean SHAP values corresponding to all samples per EDLOS or BT in minutes, respectively. Figure 4(a1,b1) shows the relationship between EDLOS/BT (0 ≤t≤ 24 h) and IHM in older patients; when the EDLOS < 5 h or BT ≥ 3 h, there was a higher risk of IHM. Furthermore, we defined EDLOS and BT in hours and calculated the median [IQR] of SHAPs as shown in Supplementary Figure S2, which shows the effect of the varying EDLOS and BT (in hours) on IHM. The influence trends of EDLOS on IHM were similar with the increase of EDLOS, SHAP values moved from the positive range to the negative range, and the trend became more obvious with the increase in age (see Figure 4 and Figure S2). For example, for the age ≥90 group, with the increase of EDLOS, the median [Q1, Q3] of SHAP values first decreased from a positive contribution (e.g., 0.103 [0.060, 0.171] at 2–4 h) to 0 (in about 5 h) and then became a negative contributor (indicating a decreased risk of IHM, e.g., −0.110 [−0.177, −0.084] at 14–16 h). The effect of EDLOS and BT with IHM is highly volatile, but we can still observe an obvious trend from the perspective of big data: lower EDLOS and higher BT were associated with increased risk of IHM.

**Figure 5:**
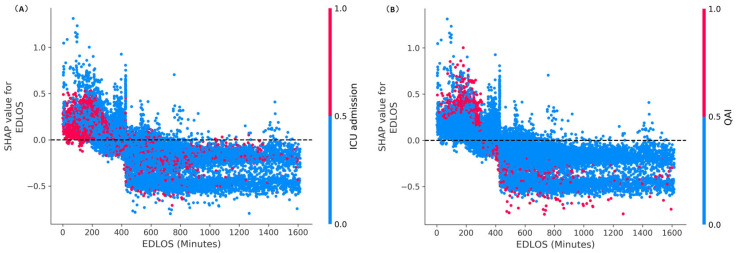
SHAP dependence plots of EDLOS with the ICU admission (**A**) and QAI (quality assurance issue) (**B**) interaction in the older population. The higher the SHAP value of a feature, the higher its impact on the risk of IHM. Each dot represents a patient. Dots are colored by the feature value for that person and piled up vertically to show density. Particularly, for binary ICU and QAI variables (i.e., {0,1}), the red dot represents a value of 1, and the blue represents a value of 0. QAI, quality assurance issue; EDLOS, length of stay in the emergency department.

**Figure 6:**
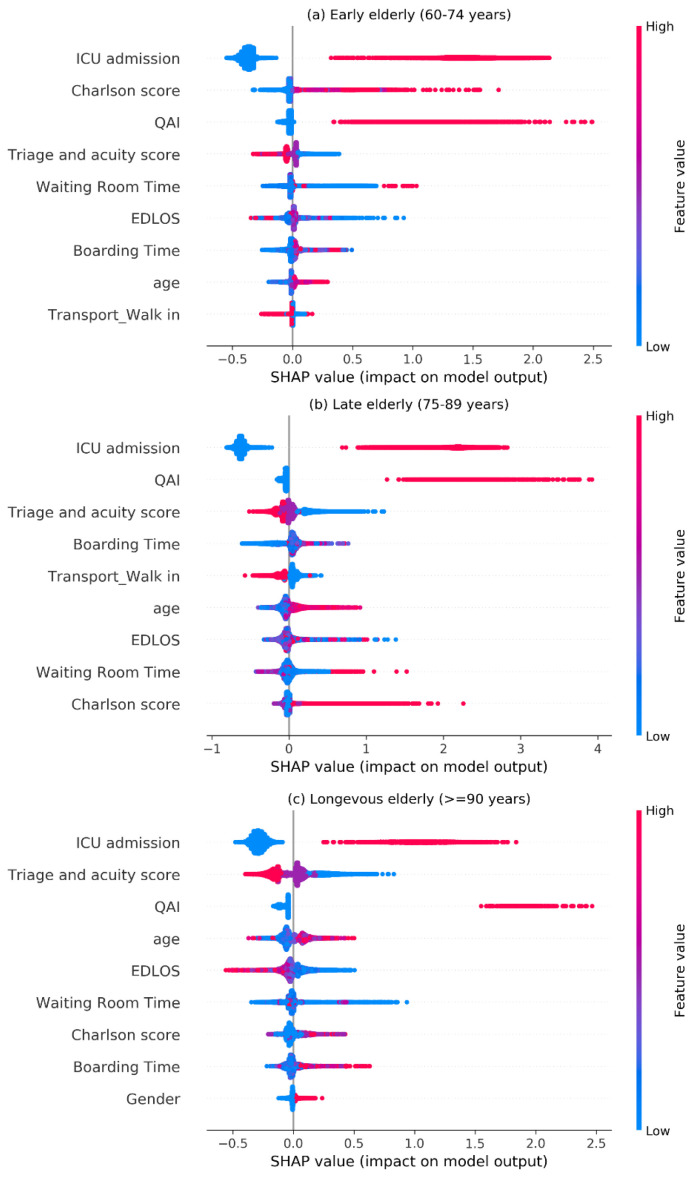
SHAP visualization for top nine features of the IHM (in-hospital mortality) in three age groups. The higher the SHAP value of a feature, the higher its impact on the risk of IHM. Each dot represents a patient. Dots are colored by the feature value for that person and piled up vertically to show density. Particularly, for binary variables (i.e., {0,1}), the red dot represents a value of 1, and the blue represents a value of 0. QAI, quality assurance issue; EDLOS, emergency department length of stay; Charlson score, Charlson Comorbidity Index.

Figure 5 illustrates the SHAP dependence plots of EDLOS with the ICU and QAI interaction in the older population, from which we can see that most ICU admissions and QAI patients were mainly concentrated in the low EDLOS (e.g., <5 h). Figure 6 shows the SHAP visualization of the top 9 risk factors for predicting IHM in each age subgroup, from which we can see that ICU admission was the most important risk predictor of IHM. QAI, the triage and acuity score, the Charlson score, EDLOS and BT were all important predictive factors of IHM.

**Figure 7:**
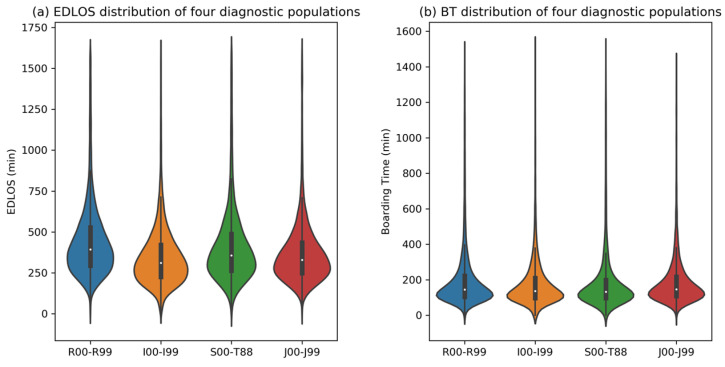
Distribution of emergency department length of stay (EDLOS, in minutes) and boarding time (BT, in minutes) of four major diagnostic populations, namely R00-R99 (symptoms, signs and abnormal clinical and laboratory findings, not elsewhere classified), I00-I99 (diseases of the circulatory system), S00-T88 (injury, poisoning and certain other consequences of external causes), and J00-J99 (diseases of the respiratory system).

**Figure 8:**
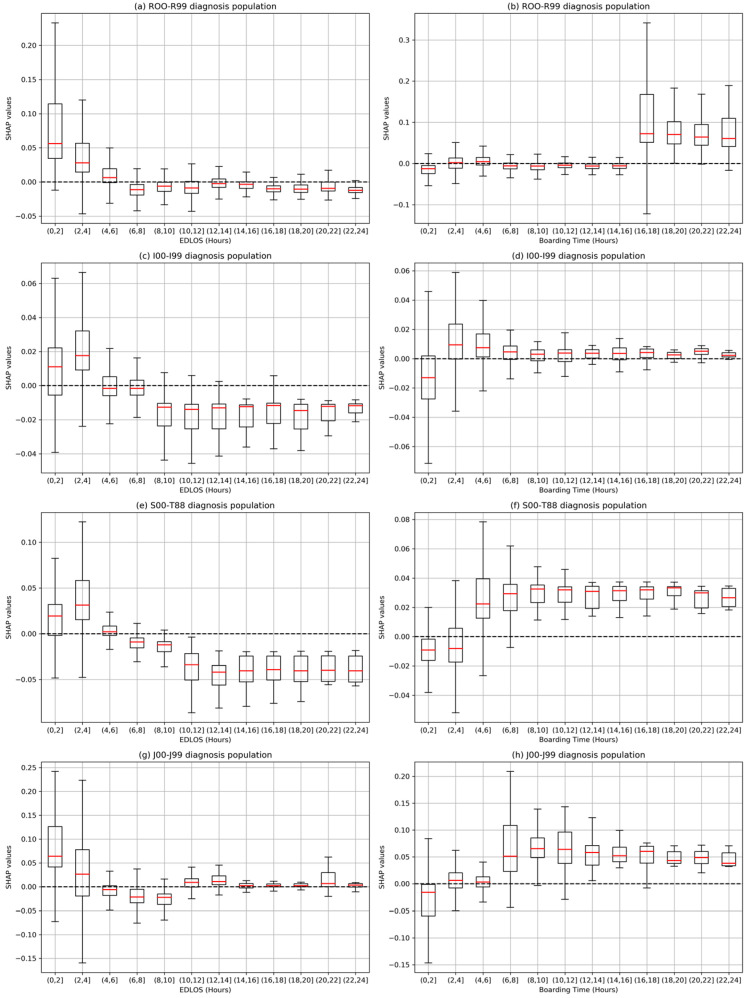
Effect of varying emergency department length of stay (EDLOS, in hours) and boarding time (BT, in hours) on in-hospital mortality (IHM) for four major diagnostic populations based on the SHAP method, the R00-R99 (symptoms, signs and abnormal clinical and laboratory findings, not elsewhere classified), the I00-I99 (diseases of the circulatory system), the S00-T88 (injury, poisoning and certain other consequences of external causes), and the J00-J99 (diseases of the respiratory system). The box plots report the median and the interquartile range of the SHAP values of patients within the range of EDLOS and BT.

Supplementary Table S5 shows the distribution of 21 major diagnostic categories. Four major diagnostic categories with mortality rates exceeding 10%, the R00-R99 (20.7%, symptoms, signs and abnormal clinical and laboratory findings, not elsewhere classified), the I00-I99 (28.5%, diseases of the circulatory system), the S00-T88 (10.7%, injury, poisoning and certain other consequences of external causes), and the J00-J99 (13.5%, diseases of the respiratory system). Figure 7 shows the distribution of EDLOS and BT for these four diagnostic subgroups. Figure 8 shows the effect of varying EDLOS and BT (in hours) on IHM for four major diagnostic populations based on the SHAP method, from which we can see that the risk trends of EDLOS and BT on IHM in the four diagnostic subgroups are similar and confirm that lower EDLOS and higher BT have a higher IHM risk. However, different subgroups would have different cutoff values, for example, for the I00-I99 diagnosis subgroup, when BT exceeds 2 h, it has a significant positive effect on the risk of IHM; however, for the S00-T88 diagnosis subgroup, BT shows a higher risk of IHM after more than 4 h.

## 4. Discussion

Overcrowding can increase EDLOS, which is used by hospital administrators as an indicator of the quality of care delivered in the ED. However, the relationship between the EDLOS and IHM remains unclear and underestimated, and advanced age is an established independent risk factor for IHM. In this study, machine learning methods were used to identify the specific EDLOS and BT associated with increased IHM following ED care of older patients. By quantifying the predictive importance of different EDLOS to IHM, our study showed that lower EDLOS and higher BT were significantly associated with a higher risk of IHM in older patients.

In previous studies, the main method used was Logistic Regression to analyze the relationship between EDLOS and IHM [8]. Since tree-based models consistently outperform standard deep models on tabular-style datasets in many medical applications [16], three other tree-based predictive models (i.e., Random Forest, XGBoost, and LightGBM) were chosen for the comparison. Due to the faster training speed and better accuracy, the highly efficient LightGBM model was used as the final predictive classifier. In addition, the LightGBM model can handle missing values, and the optimal null value splitting direction is obtained by automatic learning based on improvement in training performance. On that basis, the SHAP method [16,17,18] was used to interpret the LightGBM classifier results to analyze the importance of individual features. The SHAP method not only provides local interpretation of inference data, enabling users to analyze key factors that are positively or negatively affecting the model’s decision-making process, but also provides global interpretation, especially from the collective feature importance plots (see Figure 4, Figure 5 and Figure S5).

Although some studies had explored the relationship between EDLOS and IHM, no consistent conclusion had been reached. Some studies realized in different countries have found no specific EDLOS cutoff [19,20,21] and the cutoffs were not uniform among the studies that reported them, such as 1.2 h [22], 1.5 h [23], 2 h [24], 3 h [25], 4 h [26,27,28], 5 h [29], 6 h [30], 8 h [31,32], 12 h [33], and 24 h [34]. In our meta-analysis, we found that low EDLOS increases IHM in non-ICU admitted patients, whatever the age (8). For the three age subgroups, the cutoff (i.e., SHAP value = 0) of EDLOS increased with age, for example, as shown in Figure 4, the approximate cutoffs of early elderly, late elderly, and longevous elderly populations are 3 h, 4 h, and 5 h, respectively, and it seems that high EDLOS is less deleterious in longevous elderly patients than in early and late elderly patients. There is a positive correlation between the risk of IHM and BT, and long BT may have a significant adverse increase of IHM in the older population. In addition, we further analyzed four diagnostic subgroups with high IHM levels (see Figure 7), where lower EDLOS and higher BT are significantly associated with the risk of IHM, and the specific cutoff values will vary depending on the different diagnosis (see Figure 8).

Through the SHAP interpreter method, each patient’s EDLOS or BT feature (in minutes) corresponds to a SHAP value (like the logarithmic of estimate odds ratio). Because of the complexity of ED admission patients, the SHAP trends have a strong volatility with the increase of EDLOS and BT for each age population. Lower EDLOS (e.g., <4 h) had a more significant increase of IHM (see Figure 4 and Figure 8), where the flagged rate of QAI was higher than in high EDLOS (e.g., 9.96% in ≤1 h vs. 1.84% in 7 h <t≤ 8 h). Therefore, we can speculate that older patients may benefit more from long ED care rather than an accelerated admission. In addition, a clear trend was observed regarding higher BTs having a stronger positive correlation with the risk of IHM in both subgroups (see Figure 4 and Figure 8). Singer et al. [35] have already demonstrated that mortality is increased in boarding patients whatever the age in the ED. That is, under similar EDLOS, longer BT would adversely affect patient outcomes.

The top-ranking risk predictors of IHM were different among the age groups 60–74, 75–89, and ≥90 years (see Figure 6). The role of risk factors obtained by the SHAP method followed medical common sense. The Charlson comorbidity score has a stronger predictive effect; the higher the Charlson score is, the greater the corresponding SHAP value shows an increased risk of IHM. Patients with severe acuity triage scores from level one to level three showed the highest SHAP value related to a higher risk of IHM. Negative SHAP values indicating a decreased risk of IHM were obtained for patients who entered the ED in a walk-in transport mode. Moreover, patients experiencing QAI had a higher risk of IHM. Our results should be taken into account by hospital policymakers to propose that EDs should be redesigned to include specific areas allowing adequate monitoring of patients with specific teams focused on their care management and to ensure that the best care will be delivered when they stay longer in the ED before admission to the hospital. Moreover, ED healthcare teams must be cautious when deciding to admit a patient to the wards after a short EDLOS in any age group. From our study using artificial intelligence minimizes heterogeneity and allows to understand the precise role of the time spent in the ED on the quality and safety of the care delivered to an heterogeneous population managed in the ED. Many guidelines emphasize the importance of the time during the care process of acute diseases from the prehospital to the ED setting (e.g., “time is brain”, “time is heart”…).

This study has some limitations. First, although we included a large ED cohort observed for seven years (2010–2016), these data do not include recent years, especially the COVID-19 pandemic period. Second, we mainly used the timestamp information related to the length of stay in the ED (such as triage registration time, the start of care time, admission decision time, and ED exit time) and we did not include the entire EMR (e.g., lab tests and treatments), which may further increase the predictive performance. Third, this study is only a retrospective study. A prospective study needs to be designed in the future, in which the evaluation of the ED throughput process needs more attention. Fourth, based on the World Health Organization criteria for the classification of older persons (age ≥60 years), the older populations were further divided into the early elderly (60–74 years), the late elderly (75–89 years), and the longevous elderly (over 90 years). However, certain developed countries in the West have chosen age 65 as the cutoff point of older patients. Finally, although our results were statistically significant, they only reflect the population of one academic medical center. A multicenter comparative study involving hospitals in different countries or different types of hospital (such as community hospitals) is needed to demonstrate the robustness and relevance of our results, which will provide strong suggestions for policymakers to propose new older patients ED processes of care.

## 5. Conclusions

This is the first study analyzing a large EMR dataset using machine learning methods to determine the relationship between EDLOS and IHM in older patients. Our study confirms that lower EDLOS and higher BT are correlated with IHM in older patients. ED healthcare providers can improve the care process for patients who will stay a short time in the ED, but they do not have significant impact on the hospital beds availibity. Policymakers, administrators, and ED leaders should propose new procedures to reduce BT and provide dedicated well-trained ED professionals, including special elderly patient care areas in the ED.

## Figures and Tables

**Table 1:** Characteristics of the study population.

Characteristics	Older Patients, Age ≥60 (n = 78,847)	Early Elderly, Age 60–74 (n = 38,817)	Late Elderly, Age 75–89 (n = 32,261)	Longevous Elderly, Age ≥ 90 (n = 7769)	*p* Value
Age, years, median (Q1, Q3)	75 (67, 84)	67 (63, 70)	82 (78, 85)	92 (91, 95)	<0.001
Male, n (%)	37,211 (47.2)	20,052 (51.7)	14,535 (45.1)	2624 (33.8)	<0.001
Race, n (%)					<0.001
Unknown	1428 (1.8)	654 (1.7)	624 (1.9)	150 (1.9)	
White	57,429 (72.8)	26,966 (69.5)	24,167 (74.9)	6296 (81)	
Black	11,675 (14.8)	6658 (17.2)	4205 (13)	812 (10.5)	
Hispanic	3032 (3.8)	1847 (4.8)	1079 (3.3)	106 (1.4)	
Asian	2497 (3.2)	1240 (3.2)	1077 (3.3)	180 (2.3)	
Other	2786 (3.5)	1452 (3.7)	1109 (3.4)	225 (2.9)	
Language-English, n (%)	68,213 (86.5)	34,919 (90)	26,809 (83.1)	6485 (83.5)	<0.001
Insurance, n (%)					<0.001
Unknown	3,970 (5)	2147 (5.5)	1494 (4.6)	329 (4.2)	
Medicare	54,441 (69)	20,920 (53.9)	26,750 (82.9)	6771 (87.2)	
Medicaid	106 (0.1)	72 (0.2)	25 (0.1)	9 (0.1)	
Other	20,330 (25.8)	15,678 (40.4)	3992 (12.4)	660 (8.5)	
Triage and acuity score, n (%)					<0.001
Resuscitation	12,565 (15.9)	5870 (15.1)	5323 (16.5)	1372 (17.7)	
Emergent	37,963 (48.1)	18,430 (47.5)	15,837 (49.1)	3696 (47.6)	
Urgent	28,095 (35.6)	14,396 (37.1)	11,013 (34.1)	2686 (34.6)	
Less urgent	217 (0.3)	117 (0.3)	86 (0.3)	14 (0.2)	
Nonurgent	7 (0)	4 (0)	2 (0)	1 (0)	
Mode of transport, n (%)					<0.001
Unknown	2312 (2.9)	1127 (2.9)	962 (3)	223 (2.9)	
Walk-in	32,888 (41.7)	18,863 (48.6)	11,953 (37.1)	2072 (26.7)	
Ambulance	43,215 (54.8)	18,592 (47.9)	19,178 (59.4)	5445 (70.1)	
Helicopter	340 (0.4)	178 (0.5)	143 (0.4)	19 (0.2)	
Other	92 (0.1)	57 (0.1)	25 (0.1)	10 (0.1)	
ED waiting time, min, median (Q1, Q3)	10 (6, 29)	11 (6, 33)	10 (5, 27)	9 (5, 23)	<0.001
Length of stay in ED, min, median (Q1, Q3)	366 (271, 495)	370 (272, 503)	360 (269, 488)	363 (272, 487)	<0.001
Boarding time, min, median (Q1, Q3)	143 (104, 219)	145 (104, 223)	141 (103, 214)	144 (105, 218)	<0.001
Charlson score, n (%)					<0.001
0	54,001 (68.5)	26,713 (68.8)	21,691 (67.2)	5597 (72)	
1	16,995 (21.6)	7613 (19.6)	7632 (23.7)	1750 (22.5)	
2	4271 (5.4)	2192 (5.6)	1757 (5.4)	322 (4.1)	
>2	3580 (4.5)	2299 (6)	1181 (3.7)	100 (1.4)	
Quality Assurance Issue (QAI), n (%)	1960 (2.5)	882 (2.3)	859 (2.7)	219 (2.8)	0.001
Patient Safety Events (PSE), n (%)	129 (0.2)	53 (0.1)	60 (0.2)	16 (0.2)	0.167
ICU admission, n (%)	16,668 (21.1)	7996 (20.6)	7039 (21.8)	1633 (21)	<0.001
Death in hospital, n (%)	2975 (3.8)	1046 (2.7)	1440 (4.5)	489 (6.3)	<0.001

ED, emergency department; IQR, interquartile range.

## Data Availability

The clinical data used in this study is not publicly available and restrictions apply to its use. Open reasonable request, the amendment can be requested to the corresponding author (AB) to share the necessary data.

## Supplementary Materials

The following supporting information can be downloaded at: https://www.mdpi.com/article/10.3390/jcm12144750/s1. Table S1.Characteristics of the study emergency department participants according to IHM (survivors and non-survivors). Table S2. Characteristics of the non-ICU and ICU admitted older (≥60) ED patients. Table S3. Characteristics of survivors and non-survivors in the older ED participants according to EDLOS. Table S4. The area under the receiver operator characteristic curve (AUROC) and 95% confidence intervals in predicting in-hospital mortality (IHM). Table S5. Distribution of 21 major diagnostic categories. Figure S1. Flow chart of population classification based on diagnostic codes. Figure S2. Effect of varying emergency department length of stay (EDLOS, in hours) and boarding time (BT, in hours) on death-in-hospital (IHM) for three age groups based on the SHAP method. The box plots report the median and the interquartile range of the SHAP values of patients within the range of EDLOS.
