# Supplementary material for: The Association between Emergency Department Length of Stay and In-Hospital Mortality in Older Patients Using Machine Learning: An Observational Cohort Study
Source: J Clin Med. 2023 Jul 18;12(14):4750. doi: 10.3390/jcm12144750 (PMC10381297; doi:10.3390/jcm12144750)
Supplement: Supplementary file 1 [file jcm-12-04750-s001.zip › jcm-2444750-supplementary.pdf]

Journal of Clinical Medicine

MDPI

Supplementary Materials

Table S1. Characteristics of the study emergency department participants according to IHM (survivors and non-survivors).

Characteristics	Age 60 (n = 78,847)	
	Survivors (n = 75,872)	Non-survivors (n = 2975)
Age, years, median [IQR]	75 [67,84]	80 [70,87]
Male, n (%)	35707 (47.1)	1504 (50.6)
Race, n (%)		
Unknown	1102 (1.5)	326 (11)
White	55412 (73)	2017 (67.8)
Black	11355 (15)	320 (10.8)
Hispanic	2972 (3.9)	60 (2)
Asian	2371 (3.1)	126 (4.2)
Other	2660 (3.5)	126 (4.2)
Insurance, n (%)		
Unknown	3340 (4.4)	630 (21.2)
Medicare	52606 (69.3)	1835 (61.7)
Medicaid	103 (0.1)	3 (0.1)
Other	19823 (26.1)	507 (17)
Triage and acuity score, n (%)		
Resuscitation	11029 (14.5)	1536 (51.6)
Emergent	36895 (48.6)	1068 (35.9)
Urgent	27726 (36.5)	369 (12.4)
Less urgent	215 (0.3)	2 (0.1)
Nonurgent	7 (0)	
Mode of transport, n (%)		
Unknown	2081 (2.7)	231 (7.8)
Walk-in	32357 (42.6)	531 (17.8)
Ambulance	41082 (54.1)	2133 (71.7)
Helicopter	264 (0.3)	76 (2.6)
Other	88 (0.1)	4 (0.1)
ED waiting time, min, median [IQR]	10 [6,30.2]	6 [1,11]
Length of stay in ED, min, median [IQR]	368 [273.8,498]	289 [205,409]
Boarding time, min, median [IQR]	143 [104,219]	139 [101,209]
Charlson score, n (%)		
0	52218 (68.8)	1783 (59.9)
1	16197 (21.3)	798 (26.8)
2	4064 (5.4)	207 (7)
>2	3393 (4.5)	187 (6.3)
Quality Assurance Issue (QAI), n (%)	1258 (1.7)	702 (23.6)
Patient Safety Events (PSE), n (%)	109 (0.1)	20 (0.7)
ICU admission, n (%)	14203 (18.7)	2465 (82.9)

ED, emergency department; IQR, interquartile range. This table compares the characteristics of survivors and non-survivors in the older group (age ≥ 60). The distribution of all variables was statistically different between the survivors and non-survivors, namely p<0.001.

Table S2. Characteristics of the non-ICU and ICU admitted older (≥ 60) ED patients.

Characteristics	Non-ICU Admitted (n = 62,179)	ICU Admitted (n = 16,668)	P value
Age, years, median [IQR]	75 [67,84]	75 [67,84]	0.002
Male, n (%)	28,678 (46.1)	8553 (51.2)	<0.001
Race, n (%)			<0.001
Unknown	426 (0.7)	1002 (6)	
White	45645 (73.4)	11784 (70.7)	
Black	9534 (15.3)	2141 (12.8)	
Hispanic	2524 (4.1)	508 (3)	
Asian	1939 (3.1)	558 (3.3)	
Other	2111 (3.4)	675 (4)	
Language-English, n (%)	53617 (86.2)	14596 (87.6)	<0.001
Insurance, n (%)			<0.001
Unknown	1635 (2.6)	2335 (14)	
Medicare	43887 (70.6)	10554 (63.3)	
Medicaid	80 (0.1)	26 (0.2)	
Other	16577 (26.7)	3753 (22.5)	
Triage and acuity score, n (%)			<0.001
Resuscitation	5895 (9.5)	6670 (40)	
Emergent	30307 (48.7)	7656 (45.9)	
Urgent	25763 (41.4)	2332 (14)	
Less urgent	209 (0.3)	8 (0)	
Nonurgent	5 (0)	2 (0)	
Mode of transport, n (%)			<0.001
Unknown	1082 (1.7)	1230 (7.4)	
Walk-in	28790 (46.3)	4098 (24.6)	
Ambulance	32168 (51.7)	11047 (66.3)	
Helicopter	67 (0.1)	273 (1.6)	
Other	72 (0.1)	20 (0.1)	
ED waiting time, min, median [IQR]	12 [6,38]	7 [4,13]	<0.001
Length of stay in ED, min, median [IQR]	382 [287,512]	301 [216,420]	<0.001
Boarding time, min, median [IQR]	144 [105,221]	139 [99,211]	<0.001
Charlson score, n (%)			<0.001
0	43449 (69.9)	10552 (63.3)	
1	12632 (20.3)	4363 (26.2)	
2	3241 (5.2)	1030 (6.2)	
>2	2857 (4.6)	723 (4.3)	
Quality Assurance Issue (QAI), n (%)	423 (0.7)	1537 (9.2)	<0.001
Patient Safety Events (PSE), n (%)	55 (0.1)	74 (0.4)	<0.001
Death in hospital, n (%)	510 (0.8)	2465 (14.8)	<0.001

ED, emergency department; IQR, interquartile range. This table compares the characteristics of ICU admitted patients and non-ICU admitted patients in the older group (age ≥ 60).

Table S3. Characteristics of survivors and non-survivors in the older ED participants according to EDLOS.

Characteristics	Low EDLOS (n = 25,806)		High EDLOS (n = 53,041)	
	Survivors (n = 24,235) 93.9%	Non-survivors (n = 1571) 6.1%	Survivors (n = 51,637) 97.4%	Non-survivors (n = 1401) 2.6%
Age, years, median [IQR]	75 [67,83]	80 [70.5,87]	74 [67,84]	79 [70,87]
Male, n (%)	12218 (50.4)	807 (51.4)	23489 (45.5)	697 (49.6)
Race, n (%)				
Unknown	763 (3.1)	279 (17.8)	339 (0.7)	47 (3.3)
White	18086 (74.6)	1005 (64)	37326 (72.3)	1012 (72.1)
Black	3013 (12.4)	125 (8)	8342 (16.2)	195 (13.9)
Hispanic	820 (3.4)	23 (1.5)	2152 (4.2)	37 (2.6)
Asian	650 (2.7)	64 (4.1)	1721 (3.3)	62 (4.4)
Other	903 (3.7)	75 (4.8)	1757 (3.4)	51 (3.6)
Language-English, n (%)	21381 (88.2)	1382 (88)	44248 (85.7)	1202 (85.6)
Insurance, n (%)				
Unknown	1972 (8.1)	517 (32.9)	1368 (2.6)	113 (8)
Medicare	16123 (66.5)	803 (51.1)	36483 (70.7)	1032 (73.5)
Medicaid	35 (0.1)	1 (0.1)	68 (0.1)	2 (0.1)
Other	6105 (25.2)	250 (15.9)	13718 (26.6)	257 (18.3)
Triage and acuity score, n (%)				
Resuscitation	5810 (24)	1070 (68.1)	5219 (10.1)	466 (33.2)
Emergent	12138 (50.1)	409 (26)	24757 (47.9)	659 (46.9)
Urgent	6248 (25.8)	90 (5.7)	21478 (41.6)	279 (19.9)
Less urgent	37 (0.2)	2 (0.1)	178 (0.3)	
Nonurgent	2 (0)		5 (0)	
Mode of transport, n (%)				
Unknown	1433 (5.9)	192 (12.2)	648 (1.3)	39 (2.8)
Walk-in	8854 (36.5)	168 (10.7)	23503 (45.5)	363 (25.9)
Ambulance	13739 (56.7)	1150 (73.2)	27343 (53)	983 (70)
Helicopter	184 (0.8)	60 (3.8)	80 (0.2)	16 (1.1)
Other	25 (0.1)	1 (0.1)	63 (0.1)	3 (0.2)
ED waiting time, min, median [Q1, Q3]	7 [5, 14]	4 [0, 7]	13 [6, 48]	8 [5, 19]
Length of stay in ED, min, median [Q1, Q3]	237 [196, 270]	210 [166.5, 253]	441 [364, 567]	419 [355, 531.2]
Boarding time, min, median [Q1, Q3]	108 [84, 137]	112 [85, 144]	174 [122, 273]	201.5 [139, 302]
Charlson score, n (%)				
0	15432 (63.7)	869 (55.3)	36786 (71.2)	914 (65.1)
1	6213 (25.6)	499 (31.8)	9984 (19.3)	299 (21.3)
2	1495 (6.2)	116 (7.4)	2569 (5)	91 (6.5)
>2	1095 (4.5)	87 (5.5)	2298 (4.5)	100 (7.1)
Quality Assurance Issue (QAI), n (%)	436 (1.8)	451 (28.7)	822 (1.6)	251 (17.9)
Patient Safety Events (PSE), n (%)	32 (0.1)	7 (0.4)	77 (0.1)	13 (0.9)
ICU admission, n (%)	6876 (28.4)	1382 (88)	7327 (14.2)	1083 (77.1)

ED, emergency department; IQR, interquartile range. This table compares the characteristics of survivors and non-survivors in the low EDLOS group (EDLOS < 300 min) and the high EDLOS group (EDLOS ≥ 300 min) in older population. The distribution of all variables was statistically different between the survivors and non-survivors, namely $p < 0.001$.

Table S4. The area under the receiver operator characteristic curve (AUROC) and 95% confidence intervals in predicting in-hospital mortality (IHM).

Age population	Logistic Regression	Random Forest	XGBoost	LightGBM
Early elderly (age 60–74 years)	0.881 [0.856,0.912]	0.875 [0.854,0.909]	0.875 [0.853,0.910]	**0.892** [0.870,0.916]
Late elderly (age 75–89 years)	0.879 [0.848,0.901]	0.868 [0.834,0.890]	0.867 [0.833,0.892]	**0.886** [0.861,0.911]
Longevous elderly (age ≥ 90 years)	**0.847** [0.797,0.890]	0.839 [0.783,0.905]	0.830 [0.772,0.869]	0.838 [0.782,0.887]

Table S5. Distribution of 21 major diagnostic categories

Chapter	Code Range	Description	Survivors, Num (%)	Non-survivors, Num (%)
1	A00-B99	Certain infectious and parasitic diseases	994 (1.3)	200 (7)
2	C00-D49	Neoplasms	366 (0.5)	27 (0.9)
3	D50-D89	Diseases of the blood and blood-forming organs and certain disorders involving the immune mechanism	1412 (1.9)	50 (1.7)
4	E00-E89	Endocrine, nutritional and metabolic diseases	2636 (3.6)	70 (2.4)
5	F01-F99	Mental, Behavioral and Neurodevelopmental disorders	708 (1)	4 (0.1)
6	G00-G99	Diseases of the nervous system	986 (1.3)	27 (0.9)
7	H00-H59	Diseases of the eye and adnexa	247 (0.3)	1 (0)
8	H60-H95	Diseases of the ear and mastoid process	57 (0.1)	0 (0)
9	I00-I99	Diseases of the circulatory system	12004 (16.2)	818 (28.5)
10	J00-J99	Diseases of the respiratory system	6563 (8.8)	387 (13.5)
11	K00-K95	Diseases of the digestive system	7920 (10.7)	215 (7.5)
12	L00-L99	Diseases of the skin and subcutaneous tissue	1840 (2.5)	12 (0.4)
13	M00-M99	Diseases of the musculoskeletal system and connective tissue	2583 (3.5)	37 (1.3)
14	N00-N99	Diseases of the genitourinary system	4868 (6.6)	118 (4.1)
15	O00-O9A	Pregnancy, childbirth and the puerperium	0 (0)	0 (0)
16	P00-P96	Certain conditions originating in the perinatal period	2 (0)	0 (0)
17	Q00-Q99	Congenital malformations, deformations and chromosomal abnormalities	10 (0)	0 (0)
18	R00-R99	Symptoms, signs and abnormal clinical and laboratory findings, not elsewhere classified	23164 (31.2)	593 (20.7)
19	S00-T88	Injury, poisoning and certain other consequences of external causes	7840 (10.6)	307 (10.7)
20	V00-Y99	External causes of morbidity	0 (0)	0 (0)
21	Z00-Z99	Factors influencing health status and contact with health services	20 (0)	0 (0)

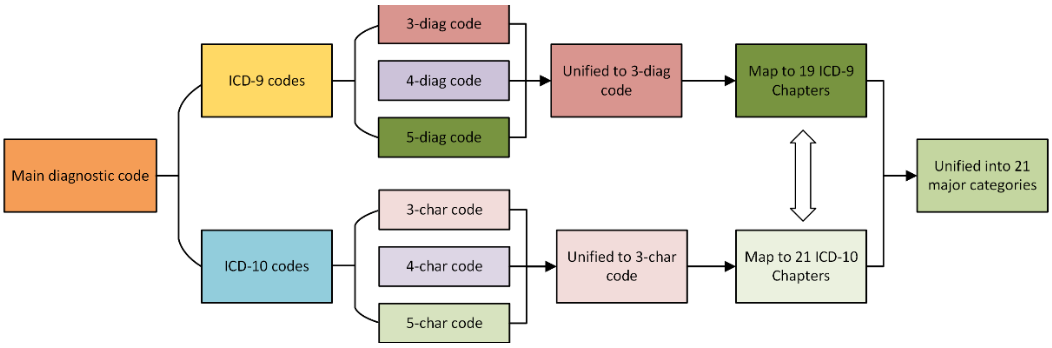

Figure S1. Flow chart of population classification based on diagnostic codes.

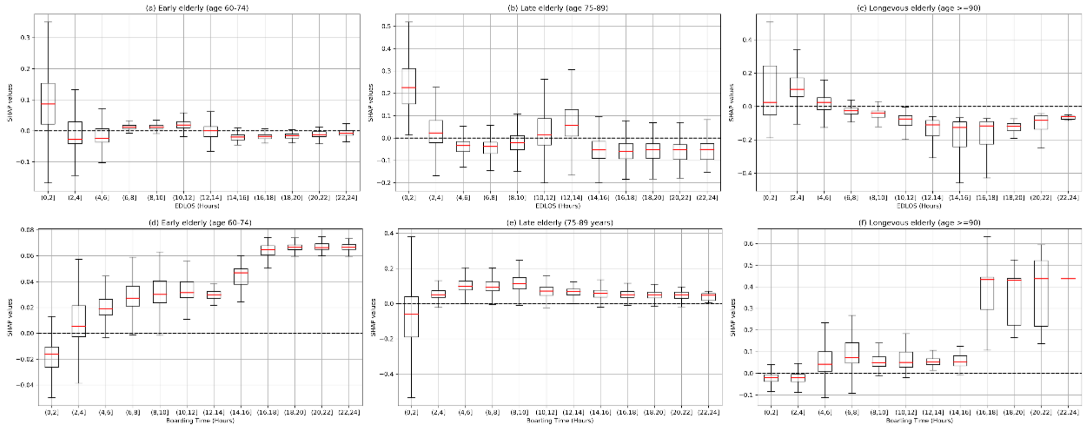

Figure S2. Effect of varying emergency department length of stay (EDLOS, in hours) and boarding time (BT, in hours) on in-hospital mortality (IHM) for three age groups based on the SHAP method. The box plots report the median and the interquartile range of the SHAP values of patients within the range of EDLOS and BT.
